# Suppression of Slit3 induces tumor proliferation and chemoresistance in hepatocellular carcinoma through activation of GSK3β/β-catenin pathway

**DOI:** 10.1186/s12885-018-4326-5

**Published:** 2018-06-01

**Authors:** Lui Ng, Ariel K. M. Chow, Johnny H. W. Man, Thomas C. C. Yau, Timothy M. H. Wan, Deepak N. Iyer, Virginia H. T. Kwan, Ronnie T. P. Poon, Roberta W. C. Pang, Wai-Lun Law

**Affiliations:** 1 Department of Surgery, Li Ka Shing Faculty of Medicine, The University of Hong Kong, Pok Fu Lam, Hong Kong; 2 Centre for Cancer Research, Li Ka Shing Faculty of Medicine, The University of Hong Kong, Pok Fu Lam, Hong Kong

**Keywords:** Slit3, β-catenin, GSK3β, Chemoresistanc, Sorafenib, Oxaliplatin, 5-FU

## Abstract

**Background:**

It is essential to understand the mechanisms responsible for hepatocellular carcinoma (HCC) progression and chemoresistance in order to identify prognostic biomarkers as well as potential therapeutic avenues. Recent findings have shown that SLIT3 appears to function as a novel tumor suppressor gene in various types of cancers, yet its clinical correlation and role in HCC has not been understood clearly.

**Methods:**

We determined the transcript levels of Slit3 in tumor and adjacent normal tissues within two cohorts (*N* = 40 and 25) of HCC patients, and correlated the gene expression with the clinicopathological data. Subsequently, the functional effects and underlying molecular mechanisms of Slit3 overexpression and/or repression were studied using cell-line and mouse models.

**Results:**

Our results demonstrated a repression in Slit3 expression in nearly 50% of the HCC patients, while the overall expression of Slit3 inversely correlated with the size of the tumor in both cohorts of patients. Stable down-regulation of Slit3 in HCC cell-lines induced cell proliferation in vitro and tumor growth in vivo, while stable Slit3 overexpression repressed these effects. Molecular investigations showed that the stable Slit3 repression-induced cell proliferation was associated with a higher expression of β-catenin and a repressed GSK3β activity. Moreover, Slit3-repression induced chemoresistance to sorafenib, oxaliplatin and 5-FU through impairment of β-catenin degradation and induction of cyclin D3 and survivin levels. The effects induced by stable Slit3-repression were diminished by transient repression of β-catenin by siRNA approach.

**Conclusion:**

This study suggests that Slit3 acts as a tumor suppressor in HCC by repressing the tumor growth and thus tumor progression. Low Slit3 level indicates a poor response of HCC cells to chemotherapy. Restoration or overexpression of Slit3 is a potential therapeutic approach to repress the tumor growth and enhance the efficacy of chemotherapeutic agents.

**Electronic supplementary material:**

The online version of this article (10.1186/s12885-018-4326-5) contains supplementary material, which is available to authorized users.

## Background

Hepatocellular carcinoma (HCC) is the second leading cause of cancer death worldwide [1]. Most HCC patients die from locally advanced or metastatic disease in a relatively short period of time, and the mechanisms responsible for HCC progression and metastasis remain a major challenge to researchers in this field. It is well-believed that the elucidation of molecular mechanisms involved in HCC progression and metastasis is important for the identification of prognostic biomarkers as well as therapeutic targets. This study will demonstrate the role of a secretory protein involved in the Slit/Roundabout (Robo) signaling pathway, namely Slit3, in HCC development and progression.

The Slit family of guidance cues interacts with the Robo family of transmembrane receptors in a wide variety of physiological processes requiring cell migration [2]. They were first identified as an important regulator in axon guidance and cell migration in Drosophila and vertebrates [3]. Three Slit proteins (Slit1, 2 and 3) have been identified so far. While the expression of Slit1 is confined to neurons, Slit2 and Slit 3 are widely expressed in mammalian tissues [4] and their deregulations have been identified in malignant tissues. Slit2 is frequently inactivated in human cancers including lung cancer [5], breast cancer [6, 7], colorectal cancer [8], ovarian cancer [9], glioma [10] and HCC [11, 12] and its tumor suppressive role that inhibits cancer cell invasion and migration [10, 13–17], angiogenesis [18, 19] and growth [8, 20–22], has been well-studied. In conjunction, hypermethylation and subsequent down-regulation of Slit3 has been reported in several cancers, including thyroid cancer, colorectal cancer, gastric cancer, nasopharyngeal carcinoma, cervical cancer, ovarian cancer and pancreatic ductal adenocarcinoma [23–32]. Importantly, Slit3 has been shown to suppress tumor growth of breast cancer in a mouse model [33] and impair cancer cell invasion and migration [24, 28, 34] through modulation of the expressions of E-cadherin, Vimentin, MMP2 and MMP9 [28].These findings demonstrate the tumor suppressive role of Slit3 in multiple types of tumor, although a comprehensive analysis addressing the clinicopathological and functional significances of Slit3 is still lacking.

Subsequently, this study aims to investigate the clinicopathological association and biological significance of Slit3 in HCC.

## Methods

### Patients and specimens

Fresh tumor specimens were collected from patients who underwent surgical resection of primary HCC at the Department of Surgery, Queen Mary Hospital, The University of Hong Kong. The study was approved by Institutional Review Board and the patients were well-informed and consented prior to inclusion.

### RNA extraction, cDNA synthesis and quantitative real-time polymerase chain reaction

To minimize the influence of heterogeneous expression of Slit3 in different regions of the tumor, tissue sections from several parts of the tumor and adjacent non-tumor liver were homogenized together, and used for RNA extraction. Total RNA was extracted using Trizol reagent and Purelink® RNA mini kit (Life Technologies, Carlsbad, CA) as previously described [35]. Total RNA (2.0 μg) was reverse-transcribed with SuperScriptII RT-PCR kit (Invitrogen, Carlsbad, CA) in accordance with the instructions of the manufacturer. Real-time PCR was performed in a final volume of 15 μl containing 1 μl RT transcript, 0.2 μM of each primer, 1X ROX reference dye and 7.5 μl of FastStart Universal SYBR Green Master (ROX) (Roche Diagnostics, Switzerland, Basel). A no RT transcript control was included for each gene to ensure the signal was truly driven by target gene amplification. The primer sequences used are: Slit3-Forward Primer: 5′- AGCGCCTTGACCTGGACA -3, Slit3-Reverse Primer: 5′- TCGGCGTGCTCTGGAAAA -3′; Actin-Forward Primer: 5′- CGAGCATCCCCCAAAGTT-3′; Actin-Reverse Primer: 5′- GCACGAAGGCTCATCATT-3′. Real-time PCR was carried out using the ABI 7900HT Fast Real-Time PCR System (Applied Biosystems, Foster, CA) at 95 °C for 10 min, followed by 40 cycles at 95 °C for 15 s and at 56 °C for 1 min. Each assay was done in triplicate. The expression level of target mRNA was normalized to the expression of actin within the same tissue.

### Slit3 over-expression plasmid construction

Slit3 full-length coding sequence was amplified from PLC cell-line RNA by Platinum® *Taq* DNA Polymerase High Fidelity (Life Technologies) using Slit3-*Hind*III-Forward Primer 5’-CCCAAGCTTATGGCCCCCGGGTGGGCA-3′ and Slit3-*Xba*I-Reverse Primer 5’-CTAGTCTAGATTAGGAACACGCGAGGCAG-3′. The PCR cycle was 94 °C for 2 min, followed by 30 cycles of 94 °C for 30 s, 56 °C for 30 s and 68 °C for 5 min. The Slit3 PCR product was purified by Qiagen PCR purification system (Valencia, CA, USA), digested with *Hind*III and *Xba*I restriction enzymes (New England Biolabs), ligated within these restriction sites in the pcDNA3.1 vector by DNA ligase (New England Biolabs) and transformed into DH5α competent cells (Life Technologies) according to the manufacturer’s instructions. The plasmids were extracted by the Qiaprep® Spin Miniprep Kit (Qiagen) and the sequence fidelity was confirmed by Sanger sequencing.

### Cell lines, tissue culture, transfections and reagents

HCC cell lines LM3, PLC, Hep3B, 97 L, HepG2 and Huh7 were cultured in DMEM medium supplemented with 10% heat-inactivated FBS, 5 U/ml penicillin and 50 μg/ml streptomycin (Life technologies), at 37 °C in a fully humidified atmosphere of 5% CO_2_ and were passaged according to the manufacturer’s recommendations. Plasmids for stable knockdown of Slit3 were purchased commercially (Origene). The shRNA sequence was synthesized as per the Slit3 siRNA sequence prescribed by us (sense: CGCGAUUUGGAGAUCCUUAtt; anti-sense: UAAGGAUCUCCAAAUCGCGca) and was cloned into a pGFP-V-RS vector downstream to the U6 promoter by the manufacturer. Stable transfections of Slit3-shRNA and the negative control plasmid (Origene) into LM3 and PLC cells were performed using Lipofectamine 2000 reagent (Invitrogen) following puromycin selection. Stable transfections of pcDNA-Slit3 and the vector control into Hep3B cells were performed using Lipofectamine 2000 reagent (Invitrogen) following G418 selection. Transient transfections of siRNA against β-catenin (Invitrogen) and control siRNA were performed using Lipofectamine 3000 reagent (Invitrogen).

### Protein extraction and western blot analysis

Protein extraction was performed by resuspending the cells in RIPA buffer (Cell Signaling Technology, Danvers, MA) containing 1 mmol/L phenoylmethylsulfonyl fluoride. Following 1 h incubation on ice and centrifugation at 14,000 x g for 10 min, the protein in supernatant was mixed with sodium dodecyl sulfate sample buffer, denatured, resolved in sodium dodecyl sulfate–polyacrylamide gel electrophoresis, and transferred to PVDF membranes (GE Healthcare, Piscataway, NJ). Antibody against Slit3 was purchased from Novus Biologicals (Littleton, CO). Antibodies against GSK3β and phospho-GSK3β (ser9) were purchased from Cell Signaling Technology. Anti-actin was from Santa Cruz biotechnology (Santa Cruz, CA). Anti-β-catenin was purchased from BD Biosciences (San Diego, CA). Protein expression levels were quantified using ImageJ software (imagej.nih.gov/ij/) and normalized to the expression of actin. Each experiment was repeated at least 3 times and representative western blots are shown in each case.

### Animal work

The protocol was approved by the Committee on the Use of Live Animals in Teaching and Research (CULATR) of The University of Hong Kong. Tumors were allowed to grow in nude mice by injection of LM3-shCTL/shSlit3 and Hep3B-pcDNA/Slit3 cells subcutaneously into flank regions with 5 × 10^6^ cells per site. Sixth week post-operation, the mice were sacrificed, and tumors were excised, measured and processed for immunohistochemical study.

### Immunohistochemical study of mice specimens

Formalin-fixed and paraffin-embedded specimens were cut into 5 μm thick sections by microtome and mounted on pre-coated slides. The sections were deparaffinized in xylene and rehydrated in serial dilutions of ethanol. Antigen retrieval was done by microwave treatment at low power for 10 min in a preheated citrate buffer. The endogenous peroxidase and biotin activity were blocked using a biotin blocking kit (Dako). The sections were then blocked with horse serum for 30 min and incubated with the primary antibodies against Slit3 (Novus Biologicals), p27 (Santa Cruz biotechnology, Santa Cruz, CA), CD31 (Abcam, Cambridge, MA), phospho-GSK3β (ser9) (Cell Signaling Technology) and β-catenin (BD Biosciences) overnight at 4 °C in a moist chamber. After incubation, slides were rinsed thrice with TBS-Tween20 and twice with TBS, and were probed with biotinylated secondary antibody (Dako) for an hour. The washing steps were repeated and the slides were then incubated with avidin-HRP (Dako) for an hour. Sites of bound antibody were visualized using liquid DAB+substrate-chromogen system (Dako) and the sections were counterstained with Gill’s Hematoxylin and mounted using DPX mountant (BDH Laboratory, UK).

### Cell viability assay

Ten thousand LM3 and PLC-shSlit3 and shCTL stable cells were seeded in a 96-well plate for 24 h and then subjected to treatment with 10 μM Sorafenib, 10 μM oxaliplatin or 100 μM 5-FU. Cell viability was assessed after 72 h of drug treatment using the MTT reagent (Life technologies).

### Statistical analysis

Data analysis was performed using SigmaStat 3.5 (Systat Software Inc., San Jose, CA, USA). Fisher exact test was used to compare clinicopathological parameters between high and low Slit3 patients. The Mann-Whitney test or student t-test was used to analyze differences between experimental groups of clinical specimens and cell-line models. Spearman’s correlation test was applied to determine correlations. A *p*-value< 0.05 was considered statistically significant.

## Results

### Frequent Slit3 mRNA repression and its association with tumor size of HCC patients

**Fig. 1:**
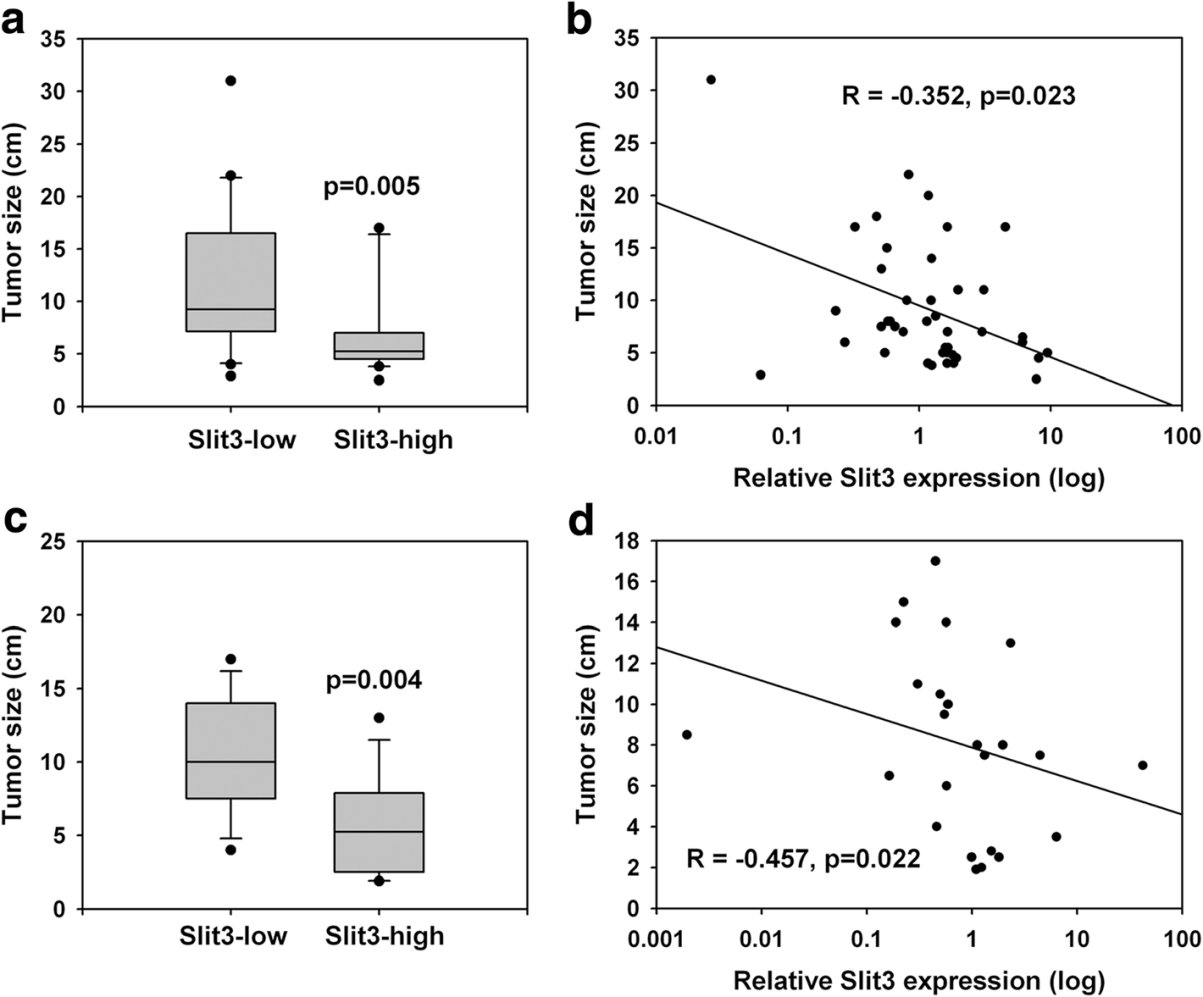
Slit3 repression correlated with HCC tumor growth. **a** Tumor size of HCC patients cohort 1 (*n* = 40) with high or low Slit3 expression. **b** Relative Slit3 expression inversely correlated with tumor size of HCC patients cohort 1 (*n* = 40). **c** Tumor size of HCC patients cohort 2 (*n* = 25) with high or low Slit3 expression. **d** Relative Slit3 expression inversely correlated with tumor size of HCC patients cohort 2 (*n* = 25)

Quantitative PCR was applied to determine the Slit3 gene expression in synchronous primary HCC tumors and the adjacent non-tumorous liver (*N* = 40). Although, no significant difference was seen in Slit3 gene expression between HCC and adjacent non-tumorous livers, we observed that 42.5% (*N* = 17) of the HCC patients showed Slit3 down-regulation (tumor/non-tumor< 1), suggesting that Slit3 repression was a frequent event associated with HCC. Subsequently, we sorted the patients into high and low Slit3 groups as per the median (tumor/non-tumor) Slit3 expression (low≤ Fold change = 1.203 > high) and compared their clinicopathological parameters (Table 1 and Additional file 1). Statistical analyses using Fisher exact test showed that Slit3 expression correlated with the HCC tumor size. Within the high Slit3 expression group, 55% (*N* = 11) of the patients had a large tumor (size > 5 cm), and this number significantly increased to 85% (*N* = 17) within the low Slit3 expression group (*p* = 0.048). Furthermore, patients with a lower relative Slit3 gene expression showed a significantly larger tumor (median: 9.25 cm) when compared with those with a higher Slit3 gene expression (median: 5.25 cm, *p* = 0.005; Fig. 1a); consequently suggesting a significant inverse correlation between Slit3 expression and tumor size (*R* = − 0.352, *p* = 0.023; Fig. 1b), indicating that Slit3 closely and inversely associated with HCC tumor growth. Although, we found that Slit3 was not affected by any other clinicopathological parameter.

**Table 1:** Clinicopathological correlation of Slit3 expression in HCC patients

		Slit3 overexpression (T/N)		*p*-value
		low	high	
Age	< 55	10	7	0.523
	> = 55	10	13	
Gender	Male	19	15	0.182
	Female	1	5	
HBV infection^a^	No	3	3	1.000
	Yes	15	16	
Prior liver-directed treatments^a^	No	17	18	0.345
	Yes	4	1	
Differentiation^a^	Well	3	5	0.682
	Moderate/Poor	11	10	
Cirrhosis^a^	No	14	10	0.313
	Yes	5	8	
Tumor Size	<=5	3	9	0.048
	> 5	17	11	
Microvascular invasion^a^	No	10	13	0.741
	Yes	8	7	
Stage^a^	1 to 2	9	11	1.000
	3 to 4	9	9	
Distant metastasis^a^	No	9	12	1.000
	Yes	8	9	

^a^In some categories, the total number of patients was less than 40 due to incomplete information

In order to validate the association between Slit3 expression and HCC tumor size, we investigated the Slit3 gene expression in another cohort of HCC patients (*N* = 25). In line with the results obtained in the first cohort, Slit3 down-regulation (tumor/non-tumor< 1) was frequently observed 56% (*N* = 14) of the patients. The median expression in the second cohort was 0.593. Within the high Slit3 expression (>Fold change = 0.593) group, 50% of the patients (*N* = 6) showed a large tumor (size > 5 cm), while the percentage significantly increased to over 90% (*N* = 12) in patients with low Slit3 expression (≤Fold change = 0.593; *p* = 0.003). Patients with a lower relative Slit3 gene expression showed a significantly larger tumor (median: 10 cm) when compared with those with a higher Slit3 gene expression (median: 5.25 cm, *p* = 0.004; Fig. 1c), indicating a significant inverse correlation between Slit3 expression and size of the HCC tumor (*R* = − 0.457, *p* = 0.022; Fig. 1d). Taken together, results from both the HCC patient cohorts showed that the expression of Slit3 in HCC inversely correlated with the size of the tumor.

### Slit3 negatively regulates cell proliferation of HCC in vitro

**Fig. 2:**
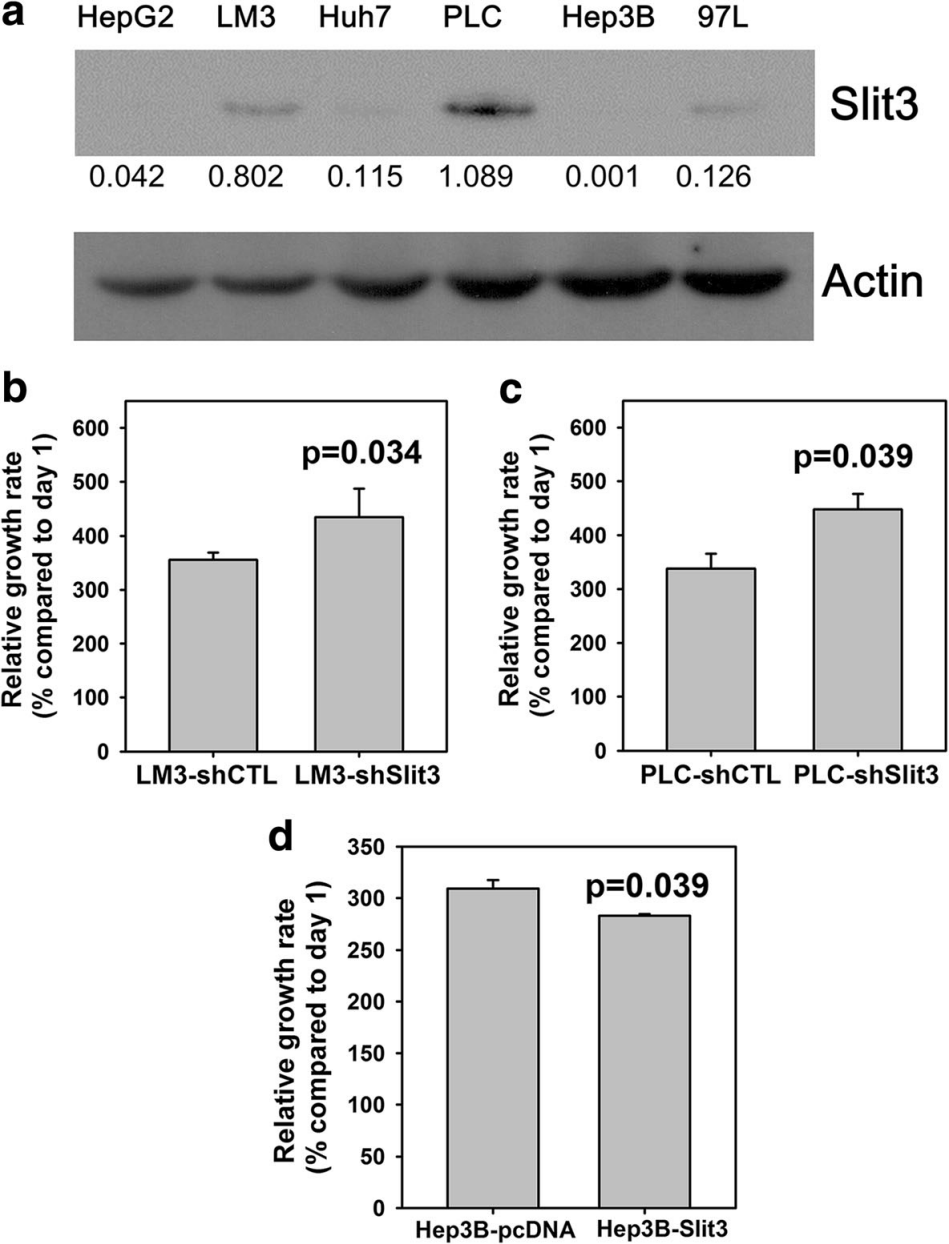
Slit3 negatively regulated HCC cell growth. **a** Expression of Slit3 in HCC cell-lines. **b** and **c** Stable Slit3 repressed clones (shSlit3) of LM3 and PLC cells displayed induced cell proliferation when compared with the control cells (shCTL) after 72 h. **d** Stable Slit3 overexpressed clones (Slit3) of Hep3B cells displayed impaired cell proliferation when compared with the control cells (pcDNA) after 72 h. The protein expression level was quantified by ImageJ software and normalized to the expression of actin. The experiment was repeated for three times and one representative blot was shown

The results from the patient samples indicated a potential tumor suppressive role of Slit3 in HCC. To test this hypothesis, we examined the functional effect of Slit3 in HCC cell-lines. The expression of Slit3 in several HCC cell-lines was determined initially (Fig. 2a). While LM3 and PLC showed a relatively higher Slit3 expression, HepG2, Huh7, Hep3B and 97 L showed a low Slit3 expression. Based on this result, we generated Slit3-downregulated stable clones from LM3 and PLC, by stable transfection of Slit3-shRNA, in order to examine the effect of Slit3 repression on cell proliferation. The relative cell number was determined by MTT in terms of absorbance and growth rate was expressed as the percentage of cell absorbance on day 3 with reference to that on day 1. As shown in Fig. 2b and c, LM3-shSlit3 and PLC-shSlit3 demonstrated a significantly higher growth rate when compared with their corresponding vector control LM3-shCTL (434.4% vs 355.9%, *p* = 0.034) and PLC-shCTL (447.4% vs 337.9%, *p* = 0.039), respectively, suggesting that Slit3 repression significantly induced HCC cell proliferation. Similarly, the effect of Slit3 overexpression on HCC cell proliferation was demonstrated by stable transfection of Slit3-expression construct in Hep3B, which expressed low level of Slit3 (Fig. 2d). Hep3B-Slit3 stable cells showed a significantly lower growth rate when compared with Hep3B-pcDNA control cells (282.9% vs 309.2%, *p* = 0.039), suggesting that Slit3 over-expression significantly reduced HCC cell proliferation.

**Fig. 3:**
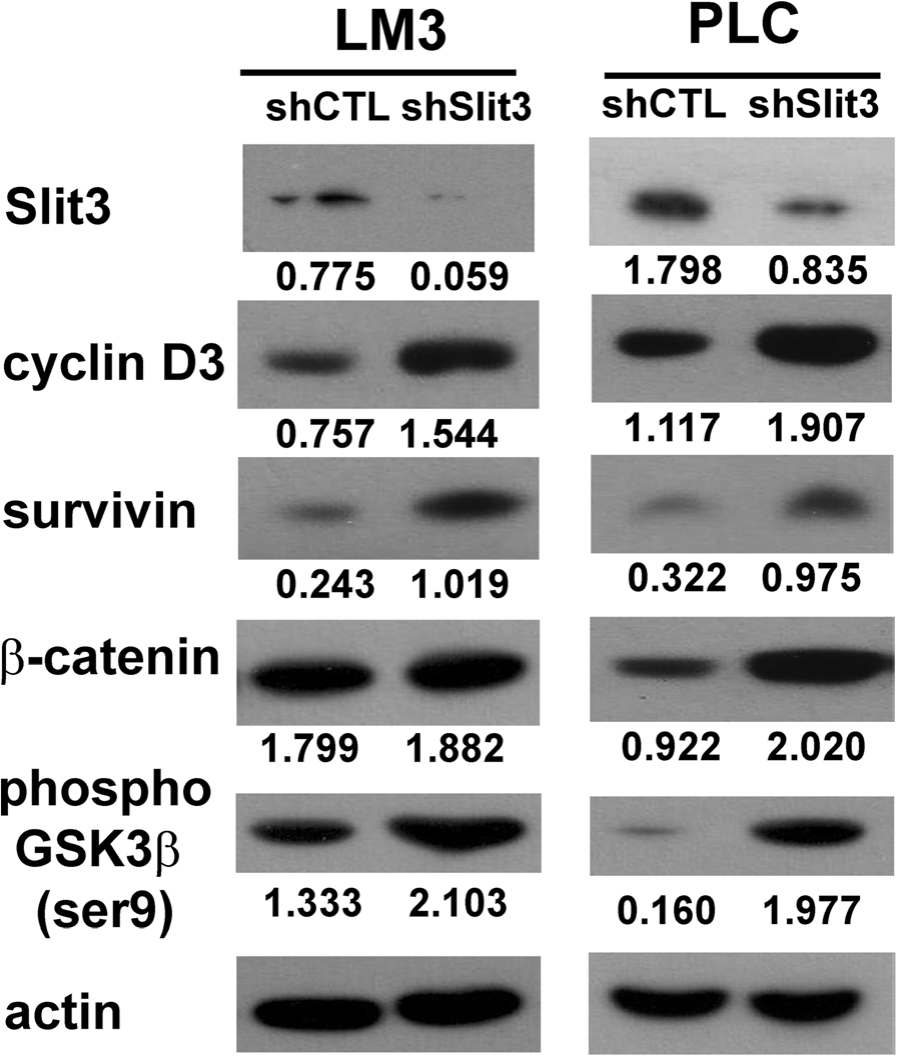
Molecular mechanism associated with stable Slit3 repression. Protein expression of certain proteins associated with cell cycle progression (cyclin D3), apoptosis (survivin) and GSK3β/β-catenin pathway (β-catenin and phosopho-GSK3β (ser9) in LM3 and PLC Slit3 shRNA transfectants (shSlit3) and shRNA control (shCTL). The protein expression level was quantified by ImageJ software and normalized to the expression of actin. The experiment was repeated for three times and one representative blot was shown

Western blot results showed that the induced cell proliferation in Slit3-repressed PLC and LM3 cells was accompanied with an induction of cyclin D3 and survivin (Fig. 3), suggesting that Slit3 down-regulation induced HCC cell growth through activation of G1/S phase transition and inhibition of apoptosis. Subsequently, we hypothesized that Slit3 regulated HCC tumor growth through manipulation of the GSK3β/β-catenin pathway which is commonly associated with the development of HCC and other liver diseases [36]. Our western blot results showed that the stable repression of Slit3 induced the expression of GSK3β (ser9) in LM3 and PLC cells (Fig. 3), indicating that GSK3β which plays an important role in β-catenin degradation, was inactivated through phosphorylation of GSK3β on ser9 residue upon Slit3 repression. Consequently, we also noted that β-catenin was induced in PLC-shSlit3 cells when compared to their shCTL cells (Fig. 3). These results showed that Slit3 repression inactivated GSK3β and thus resulting in induction of GSK3β/β-catenin pathway.

### Effect of Slit3 alteration on HCC tumor growth in vivo

**Fig. 4:**
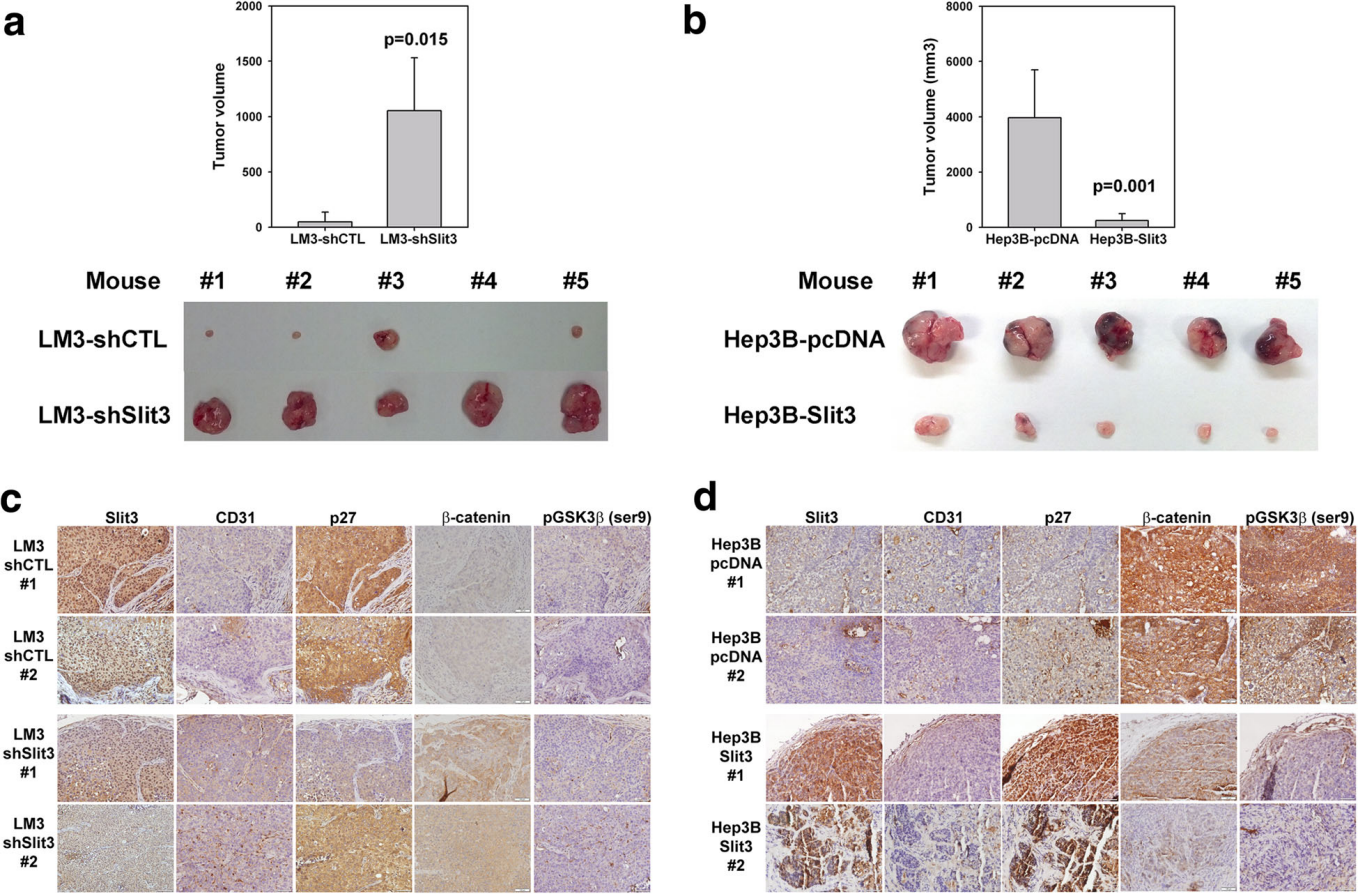
Slit3 negatively regulated HCC cell growth in vivo. **a** Stable Slit3 repressed clones (LM3-shSlit3) displayed induced tumor growth when compared with the control cells (LM-shCTL) 6 weeks post-injection of cells subcutaneously into flank region of nude mice (*n* = 5). **b** Stable Slit3 overexpressed clones (Hep3B-Slit3) displayed impaired tumor growth when compared with the control cells (Hep3B-pcDNA) 6 weeks post-injection of cells subcutaneously into flank region of nude mice (*n* = 5). **c** Immunohistochemical staining of Slit3, CD31, p27, β-catenin and phosopho-GSK3β (ser9) in tumor formed by LM3-shCTL and LM3-shSlit3 stable cells. **d** Immunohistochemical staining of Slit3, CD31, p27, β-catenin and phosopho-GSK3β (ser9) in tumor formed by Hep3B-pcDNA and Hep3B-Slit3 stable cells. Representative results from two mice of each group were shown

To investigate the effect of Slit3 repression on tumor growth in vivo, 5 × 10^6^ LM3-shCTL and LM3-shSlit3 cells were subcutaneously injected into the flank region of 5 nude mice. After 6 weeks, the tumors were excised from the mice and the tumor sizes were measured (Fig. 4a and b). In line with the in vitro effects, Slit3 repression significantly induced tumor growth in mice (1054.9 mm^3^ vs 48.2 mm^3^, *p* = 0.002). Similarly, the in vivo effect of Slit3 overexpression was examined by subcutaneous injection of Hep3B-pcDNA and Hep3B-Slit3 cells into 5 nude mice. Slit3 overexpression was found to significantly repress Hep3B tumor growth in mice when compared with Hep3B-pcDNA cells (250.5 mm^3^ vs 3975.3 mm^3^, *p* = 0.001). Taken together, these results indicated that Slit3 negatively regulated HCC tumor growth in vivo.

IHC staining was applied to determine the expression of Slit3, p27 (tumor suppressor), CD31 (vascular marker), β-catenin and phospho-GSK3β (ser9) in the tumors formed by LM3-shCTL/shSlit3 and Hep3B-pcDNA/Slit3 stable cells. The expression of Slit3 appeared weaker in LM3-shSlit3 tumors in comparison with the LM3-shCTL tumors (Fig. 4c), and stronger in Hep3B-Slit3 tumors in comparison with the corresponding Hep3B-pcDNA tumors (Fig. 4d), confirming that the Slit3 protein was indeed silenced or overexpressed in the respective tumors. The expression of CD31 was stronger in tumors developed from LM3-shSlit3 cells when compared with that from LM3-shCTL cells, and weaker in tumors developed from Hep3B-Slit3 when compared with that from Hep3B-pcDNA, suggesting that Slit3 impaired the process of angiogenesis during the tumor growth process. On the other hand, the expression of p27 which is a negative regulator of cell cycle progression [37] was lower in LM3-shSlit3 versus shCTL tumor, and stronger in Hep3B-Slit3 versus pcDNA tumor, showing that a higher expression of Slit3 suppressed cell cycle progression of HCC cells. Furthermore, β-catenin and phospho-GSK3β (ser9) were induced in LM3-shSlit3 tumors versus shCTL tumors and reduced in Hep3B-Slit3 tumors versus pcDNA tumors, suggesting that Slit3 repressed β-catenin expression by reducing the activity of GSK3β.

### Slit3 repression induces chemoresistance

**Fig. 5:**
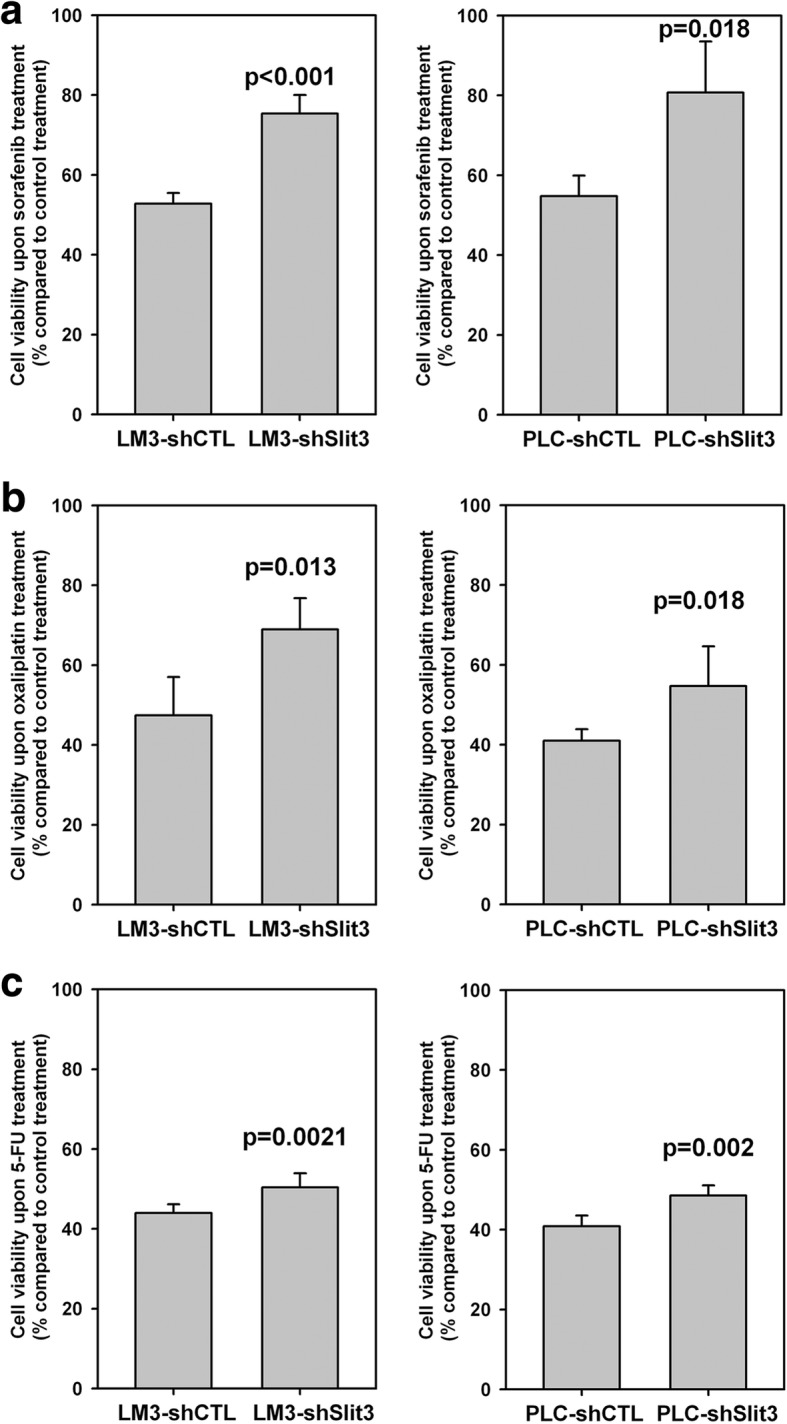
Stable repression of Slit3 expression enhanced chemoresistance in HCC cells. (Left panel) LM3-shSlit3 and shCTL cells and (right panel) PLC-shSlit3 and shCTL cells were treated with (**a**) 10 μM sorafenib, (**b**) 10 μM oxaliplatin and (**c**) 100 μM 5-FU for 72 h. The number of cells was determined by MTT assay and expressed as percentage of that under vehicle control treatment. When compared with shCTL cells, shSlit3 cells showed significantly stronger resistance to the treatment of all chemotherapeutic drugs. Each experiment was performed in triplicate and data was obtained from three independent experiments

We investigated the effect of Slit3 repression on chemoresistance of HCC cells by evaluating the growth and survival of LM3 and PLC shSlit3 cells, upon treatment of chemotherapeutic drugs including sorafenib, oxaliplatin and 5-FU for 72 h. The relative number of surviving cells were determined in terms of absorbance by MTT assay, and cell viability was expressed as the percentage of surviving cells following treatment of sorafenib/oxaliplatin/5-FU as compared to negative control. As shown in Fig. 5a to c (left panel), LM3-shSlit3 cells showed a significantly higher percentage of viable cell population than LM3-shCTL cells following exposure to sorafenib (75.35% vs 52.81%, *p* < 0.001), oxaliplatin (68.94% vs 47.44%, *p* = 0.013) and 5-FU (50.42% vs 43.96%, *p* = 0.021). Similarly, Fig. 5a to c (right panel) showed that PLC-shSlit3 cells also exhibited a significantly higher cell viability than PLC-shCTL cells following treatment with sorafenib (80.70% vs 54.75%, *p* < 0.001), oxaliplatin (54.69% vs 41.02%, *p* = 0.018) and 5-FU (48.54% vs 40.88%, *p* = 0.002). These results indicated that Slit3 suppression induced chemoresistance in HCC cells towards sorafenib, oxaliplatin and 5-FU.

**Fig. 6:**
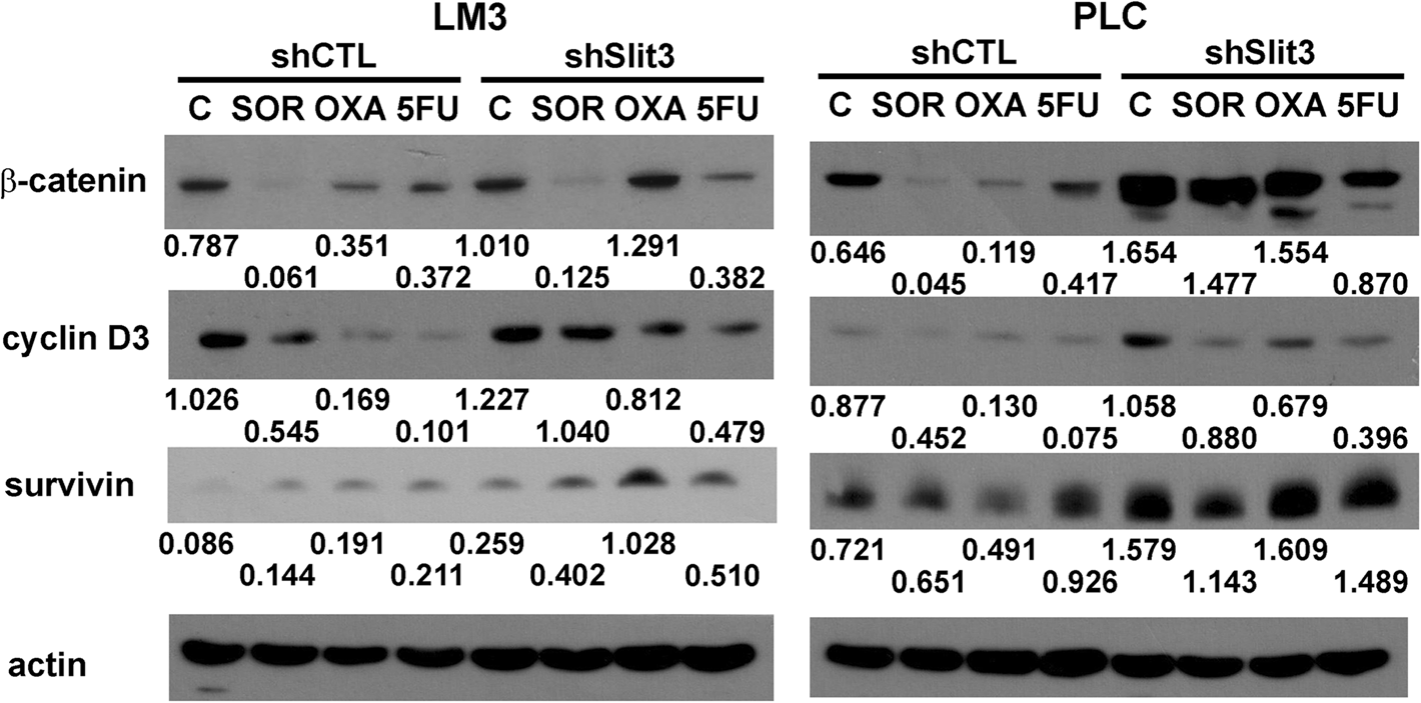
Molecular mechanism associated with Slit3 repression-induced chemoresistance. Protein expression of β-catenin, cyclin D3 and survivin in LM3 and PLC Slit3 shRNA transfectants (shSlit3) and shRNA control (shCTL) following 72-h vehicle control (C), 10 μM sorafenib (SOR), 10 μM oxaliplatin (OXA) and 100 μM 5-FU (5FU) treatment. The protein expression level was quantified by ImageJ software and normalized to the expression of actin. The experiment was repeated for three times and one representative blot was shown

Furthermore, the expression of cyclin D3 was repressed by treatment of sorafenib, oxaliplatin and 5-FU in LM3-shCTL and PLC-shCTL cells, indicating an impairment of the G1/S phase transition within the drug treated cells. Such repression was weaker in LM3-shSlit3 and PLC-shSlit3 cells (Fig. 6). On the other hand, the expression of survivin, following treatment with the chemotherapeutic agents, was stronger in LM3-shSlit3 and PLC-shSlit3 cells as compared to their respective shCTL cells; indicating that LM3-shSlit3 and PLC-shSlit3 cells were protected from the apoptotic effect induced by the chemotherapeutic agents. We previously reported that repression of β-catenin was observed in oxaliplatin-treated HCC cells [38, 39]. In this study we showed that β-catenin degradation following treatment with chemotherapeutic drugs, was impaired in Slit3-repressed LM3 and PLC cell lines. In conjuncture with the previous studies that demonstrated that the activation of β-catenin pathway enhanced the chemotherapeutic resistance of HCC cells [40, 41], results from the current study suggested that Slit3 repression contributed to the chemoresistance in HCC cells, through its inhibitory effect on β-catenin degradation.

### Down-regulation of β-catenin diminishes the effects of Slit3-repression

**Fig. 7:**
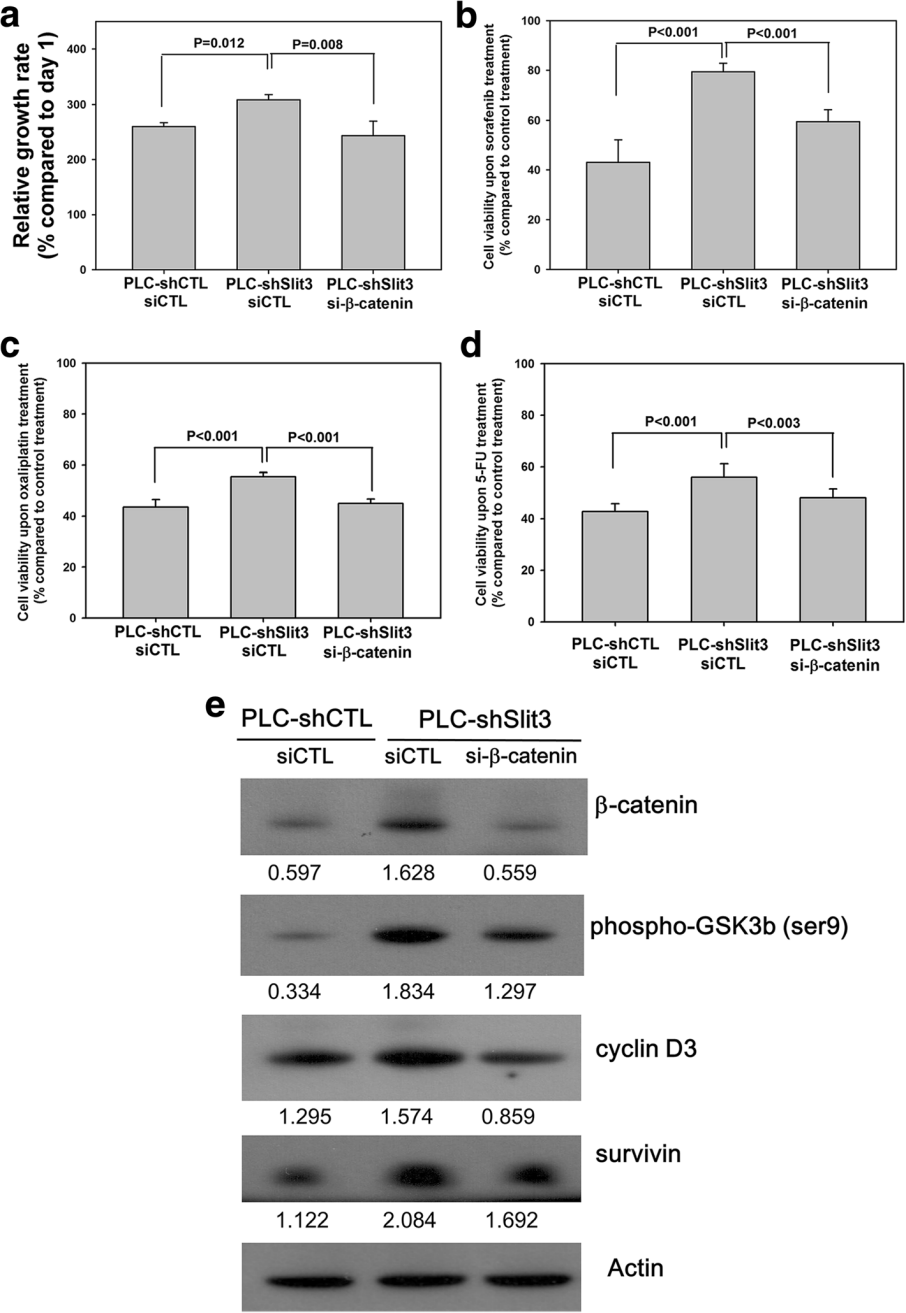
Down-regulation of β-catenin diminished the effects of Slit3-repression. Stable PLC Slit3-repressed cells (PLC-shSlit3) and control clone (PLC-shCTL) were transiently transfected with siRNA control (siCTL) or β-catenin siRNA (si-β-catenin) to investigate the effect of β-catenin down-regulation on Slit3-regulated effects. **a** PLC-shSlit3 siCTL cells showed significantly higher proliferation rate than PLC-shCTL siCTL cells whereas such induction was significantly impaired in PLC-shSlit3 si-β-catenin cells. **b** to **d** The induced chemoresistance in PLC-shSlit3 siCTL cells when compared with PLC-shCTL siCTL cells upon treatment of sorafenib, oxaliplatin and 5-FU were impaired in PLC-shSlit3 si-β-catenin cells. **e** The inductions in protein expression of phospho-GSK3β, cyclin D3 and survivin in PLC-shSlit3 siCTL cells when compared with PLC-shCTL siCTL were all reduced in PLC-shSlit3 si-β-catenin cells

In order to further study the regulatory effects of Slit3 in HCC, we repressed β-catenin expression in PLC-shSlit3 cells and investigated the downstream effects. As shown in Fig. 7a, PLC-shSlit3 cells transiently transfected with a scrambled siRNA control (PLC-shSlit3 siCTL) showed a significantly higher proliferation rate than siCTL transfected PLC-shCTL cells (308.2% vs 259.8%, *p* = 0.012), however, such an induction was significantly reduced by transient transfection of siRNA targeting β-catenin (243.3%, *p* = 0.008). Similarly, the induced chemoresistance in PLC-shSlit3 siCTL cells when compared with PLC-shCTL siCTL cells upon treatment of sorafenib (79.4% vs 43.1%, *p* < 0.001; Fig. 7b), oxaliplatin (55.4% vs 43.5%, *p* < 0.001; Fig. 7c) and 5-FU (56.0% vs 42.8%, *p* < 0.001; Fig. 7d) were also impaired by transient transfection of siRNA targeting β-catenin (59.5% for sorafenib, *p* < 0.001; 45.0% for oxaliplatin, *p* < 0.001; 48.1% for 5-FU, *p* = 0.003, Fig. 7b - d). Furthermore, following transient downregulation of β-catenin, the inductions in protein expression of phospho-GSK3β, cyclin D3 and survivin in PLC-shCTL stable cells were all reduced (Fig. 7e). These results suggested that the GSK3β/β-catenin pathway served as a key regulatory network for the Slit3-regulated effects in HCC.

## Discussion

Hepatocellular carcinoma (HCC) is the second leading cause of cancer death worldwide [1]. Poor prognosis can be attributed to tumor progression as well as a lack of promising chemotherapy for treating advanced HCC. There is hence a dire requirement of in-depth research to further elucidate the underlying molecular pathogenesis of the disease and develop therapeutic targets.

Hypermethylation and down-regulation of Slit3 has been reported in several types of cancers such as thyroid cancer, colorectal cancer, gastric cancer, nasopharyngeal carcinoma, cervical cancer, ovarian cancer and pancreatic ductal adenocarcinoma [23–32]. Additionally, Slit3 has been shown to suppress tumor growth in mouse models [33] and impairs cancer cell invasion and migration [24, 28, 34], suggesting that Slit3 functions as a tumor suppressor in a variety of cancers. In HCC, Avci et al. compared the expression of Slit3 between 8 tumor-adjacent normal and 35 tumor tissues [11]. Though they demonstrated that Slit3 was not differentially expressed, 4 of the 8 tumor-adjacent normal tissues showed Slit3 repression in the HCC tissue, suggesting that Slit3 repression was present in half of the HCC patients. In support of this interpretation, this study demonstrated that Slit3 down-regulation was present in nearly 50% of both cohorts of our HCC samples, indicating that Slit3 repression was indeed a frequent event observed in HCC. Moreover, we found that Slit3 repression inversely correlated with tumor size, which was subsequently reaffirmed in the in vitro and in vivo experiments which showed that Slit3 negatively regulated HCC cell growth. These clinical findings in combination with the functional studies suggested that Slit3 plays an important role in inhibiting tumor growth and progression in HCC.

Our next step was to investigate the molecular mechanisms associated with Slit3 repression in HCC. Several studies implicate Slit and Robo in regulating E-cadherin-dependent adhesion via the Wnt downstream signaling axis, including β-catenin and GSK3β [42], hence we investigated whether Slit3 regulated its functional effects in HCC through the GSK3β/β-catenin pathway. This study showed for the first time that Slit3 negatively regulated β-catenin expression in HCC cells, and such an induction was associated with an altered GSK3β activity (Fig. 3). Though the induction of β-catenin was not obvious in LM3-shSlit3 cells in vitro, we observed that tumors formed in vivo by LM3-shSlit3 cells showed a stronger IHC staining for β-catenin when compared with LM3-shCTL tumors (Fig. 4). The β-catenin level is regulated via phosphorylation by GSK3β in a multimeric protein complex comprising of β-catenin, GSK-3β, APC and AXIN proteins [43]. Repression of Slit3 in HCC cells inhibited the activity of GSK3β by the induced phosphorylation on the Ser 9 residue. Moreover, the effect of Slit3 alteration on tumor growth in vivo was more obvious than that on in vitro cell proliferation, which was possibly due to the effect of Slit3 on tumor angiogenesis as demonstrated by the CD31 immunohistochemical staining. We believed that Slit3 impaired angiogenesis through its regulatory effect on GSK3β/β-catenin axis which promotes angiogenesis through the activation of vascular endothelial growth factor signaling in endothelial cells [44].

For patients with advanced HCC who are not candidates for surgical resection, liver transplantation, or localized tumor ablation, systemic chemotherapy remains the mainstay of therapy. Unfortunately, HCC is a relatively chemotherapy-resistant tumor; therefore, outcomes using this mode of treatment are generally unsatisfactory. A primary area of research in HCC is the identification of biomarkers for predicting the response to chemotherapy and development of molecular targets to combat chemoresistant HCC. We tested the effect of Slit3 repression on the response of HCC cell lines to sorafenib, an oral multikinase inhibitor that executes its antitumor activities by blocking tumor angiogenesis, targeting the Raf/Mek/Erk pathway and inducing cell apoptosis [8]. Our results demonstrated that HCC cells harboring repressed Slit3 level were more resistant to sorafenib treatment. In addition, we examined the effect of Slit3 repression upon treatment with oxaliplatin and 5-FU, and our results showed that down-regulation of Slit3 induced oxaliplatin- and 5-FU chemoresistance. In this study, though the Slit3 shCTL cells showed baseline chemoresistance to all the chemotherapeutic agents, we showed that Slit3 repression, which was observed in around 50% of HCC patients of this study, was one of the contributing factors towards the development of resistance to a broad range of chemotherapeutic agents. We believe that by having a comprehensive understanding of the molecular mechanisms leading to the chemoresistant nature of HCC, novel therapeutic avenues to enhance the efficacy of chemotherapy on HCC patients can be identified and the prognosis of HCC patients can be improved.

Our study reinforces the importance of Slit3 as a therapeutic approach for HCC patients through its inhibitory effect on β-catenin pathway. The deregulation of β-catenin pathway is a hallmark of several cancers including HCC [45]. A high therapeutic efficacy of inhibiting β-catenin has already been demonstrated both in vitro and in vivo in HCC [46–49], yet there are no clinically approved anti–β-catenin agents available. Through the current study, we offer a likely advantage of applying Slit3 to inhibit β-catenin pathway as a novel treatment strategy in HCC; since Slit3 is a naturally existing protein and this might reduce the occurrence of unexpected side-effects. Indeed, the application of Slit3 as therapeutic agent has been demonstrated. A study by Denk et al. showed that the treatment with recombinant Slit3 caused a strong inhibition of migration of melanoma cells in vitro, and down-regulation of AP-1 activity [34, 50]. A different research group demonstrated that the in vitro ability of Slit3 to reduce the migratory activity of synovial cells from patients with rheumatoid arthritis and melanoma cells can be mimicked by small protein fragments derived from Slit3 containing only leucine rich repeat domain 2 [34, 50]. While the therapeutic application of a full length Slit3 may not be appropriate due to its large size that make them difficult to be expressed recombinantly, reducing their stability as well as ease of usage in in vivo studies; the recombinant Slit3 fragments offer a greater benefit for usage in cancer therapy. Within HCC, recombinant Slit3 treatment may benefit at two levels: Firstly, Slit3 can repress the growth of HCC tumor or perhaps even cause a shrinkage of the established tumor; Secondly, Slit3 could be applied as an adjuvant therapy which enhances the effectiveness of other chemotherapeutic agents such as sorafenib, oxaliplatin and 5-FU, as shown in our stable cell-line models. Though the in vitro tumor suppressive effect of Slit3 overexpression in HCC cells was not very strong, but as we observed that the negative regulatory effect of Slit3 on tumor growth was much more obvious in the in vivo model, possibly due to its involvement in tumor angiogenesis as demonstrated by the CD31 immunohistochemical staining. Based on the results from this study, we strongly believe that administration of recombinant Slit3 is a novel potential therapeutic approach for the treatment of HCC, however further investigations are necessary in order to elucidate its potency and efficacy in patients with HCC.

## Conclusion

This study showed that Slit3 was a potential tumor suppressor in HCC. Slit3 was frequently down-regulated in HCC tumor tissue and its expression inversely correlated with tumor size. Stable Slit3 repression induced the growth of HCC cells in vitro and in vivo, and induced chemoresistance to oxaliplatin, 5-FU or sorafenib, through the negative regulatory effect on β-catenin expression. Slit3 down-regulation in HCC might indicate a poor response of the tumor cells to chemotherapy, and subsequently, treatment with recombinant Slit3 is a novel potential therapeutic approach in patients with HCC, as well as other cancer types where Slit3 is repressed.

## Additional file

Additional file 1: **Table S1.** Slit3 expression and patient characteristics in cohort 1. **Table S2.** Slit3 expression and patient characteristics in cohort 2. (PDF 78 kb)

## Abbreviations

5-FU: 5 fluorouracil
GSK3β: Glycogen synthase kinase 3 beta
HCC: Hepatocellular carcinoma

## References

[1] Jemal A, Bray F, Center MM, Ferlay J, Ward E, Forman D (2011). Global cancer statistics. CA Cancer J Clin.

[2] Kidd T, Brose K, Mitchell KJ, Fetter RD, Tessier-Lavigne M, Goodman CS, Tear G (1998). Roundabout controls axon crossing of the CNS midline and defines a novel subfamily of evolutionarily conserved guidance receptors. Cell.

[3] Brose K, Tessier-Lavigne M (2000). Slit proteins: key regulators of axon guidance, axonal branching, and cell migration. Curr Opin Neurobiol.

[4] Dickinson RE, Dallol A, Bieche I, Krex D, Morton D, Maher ER, Latif F (2004). Epigenetic inactivation of SLIT3 and SLIT1 genes in human cancers. Br J Cancer.

[5] Tseng RC, Lee SH, Hsu HS, Chen BH, Tsai WC, Tzao C, Wang YC (2010). SLIT2 attenuation during lung cancer progression deregulates beta-catenin and E-cadherin and associates with poor prognosis. Cancer Res.

[6] Alvarez C, Tapia T, Cornejo V, Fernandez W, Munoz A, Camus M, Alvarez M, Devoto L, Carvallo P. Silencing of tumor suppressor genes RASSF1A, SLIT2, and WIF1 by promoter hypermethylation in hereditary breast cancer. Molecular carcinogenesis. 2013;52(6):475–87 10.1002/mc.21881 22315090

[7] Kim GE, Lee KH, Choi YD, Lee JS, Lee JH, Nam JH, Choi C, Park MH, Yoon JH (2011). Detection of Slit2 promoter hypermethylation in tissue and serum samples from breast cancer patients. Virchows Archiv : Int. J. Pathol.

[8] Dallol A, Morton D, Maher ER, Latif F (2003). SLIT2 axon guidance molecule is frequently inactivated in colorectal cancer and suppresses growth of colorectal carcinoma cells. Cancer Res.

[9] Dong R, Yu J, Pu H, Zhang Z, Xu X (2012). Frequent SLIT2 promoter methylation in the serum of patients with ovarian cancer. J. Int. Med. Res.

[10] Yiin JJ, Hu B, Jarzynka MJ, Feng H, Liu KW, Wu JY, Ma HI, Cheng SY (2009). Slit2 inhibits glioma cell invasion in the brain by suppression of Cdc42 activity. Neuro-Oncology.

[11] Avci ME, Konu O, Yagci T (2008). Quantification of SLIT-ROBO transcripts in hepatocellular carcinoma reveals two groups of genes with coordinate expression. BMC Cancer.

[12] Jin J, You H, Yu B, Deng Y, Tang N, Yao G, Shu H, Yang S, Qin W (2009). Epigenetic inactivation of SLIT2 in human hepatocellular carcinomas. Biochem Biophys Res Commun.

[13] Gu JJ, Gao GZ (2015). Zhang SM: miR-218 inhibits the migration and invasion of glioma U87 cells through the Slit2-Robo1 pathway. Oncol Lett.

[14] Jiang L, Wang Y, Rong Y, Xu L, Chu Y, Zhang Y, Yao Y (2015). miR-1179 promotes cell invasion through SLIT2/ROBO1 axis in esophageal squamous cell carcinoma. Int J Clin Exp Pathol.

[15] Gohrig A, Detjen KM, Hilfenhaus G, Korner JL, Welzel M, Arsenic R, Schmuck R, Bahra M, Wu JY, Wiedenmann B (2014). Axon guidance factor SLIT2 inhibits neural invasion and metastasis in pancreatic cancer. Cancer Res.

[16] Chen WF, Gao WD, Li QL, Zhou PH, Xu MD, Yao LQ (2013). SLIT2 inhibits cell migration in colorectal cancer through the AKT-GSK3beta signaling pathway. Int J Color Dis.

[17] Schmid BC, Rezniczek GA, Fabjani G, Yoneda T, Leodolter S, Zeillinger R (2007). The neuronal guidance cue Slit2 induces targeted migration and may play a role in brain metastasis of breast cancer cells. Breast Cancer Res Treat.

[18] Yang YC, Chen PN, Wang SY, Liao CY, Lin YY, Sun SR, Chiu CL, Hsieh YS, Shieh JC, Chang JT (2015). The differential roles of Slit2-exon 15 splicing variants in angiogenesis and HUVEC permeability. Angiogenesis.

[19] Youngblood V, Wang S, Song W, Walter D, Hwang Y, Chen J, Brantley-Sieders DM (2015). Elevated Slit2 activity impairs VEGF-induced angiogenesis and tumor neovascularization in EphA2-deficient endothelium. Mol. Cancer Res.

[20] Shi R, Yang Z, Liu W, Liu B, Xu Z, Zhang Z (2014). Knockdown of Slit2 promotes growth and motility in gastric cancer cells via activation of AKT/beta-catenin. Oncol Rep.

[21] Qiu H, Zhu J, Yu J, Pu H, Dong R (2011). SLIT2 is epigenetically silenced in ovarian cancers and suppresses growth when activated. Asian Pac. J. Cancer Prev.

[22] Kim HK, Zhang H, Li H, Wu TT, Swisher S, He D, Wu L, Xu J, Elmets CA, Athar M (2008). Slit2 inhibits growth and metastasis of fibrosarcoma and squamous cell carcinoma. Neoplasia.

[23] Davidson MR, Larsen JE, Yang IA, Hayward NK, Clarke BE, Duhig EE, Passmore LH, Bowman RV, Fong KM (2010). MicroRNA-218 is deleted and downregulated in lung squamous cell carcinoma. PLoS One.

[24] Guan H, Wei G, Wu J, Fang D, Liao Z, Xiao H, Li M, Li Y (2013). Down-regulation of miR-218-2 and its host gene SLIT3 cooperate to promote invasion and progression of thyroid cancer. J Clin Endocrinol Metab.

[25] Nones K, Waddell N, Song S, Patch AM, Miller D, Johns A, Wu J, Kassahn KS, Wood D, Bailey P (2014). Genome-wide DNA methylation patterns in pancreatic ductal adenocarcinoma reveal epigenetic deregulation of SLIT-ROBO, ITGA2 and MET signaling. Int. J. Cancer.

[26] Tie J, Pan Y, Zhao L, Wu K, Liu J, Sun S, Guo X, Wang B, Gang Y, Zhang Y (2010). MiR-218 inhibits invasion and metastasis of gastric cancer by targeting the Robo1 receptor. PLoS Genet.

[27] Yu H, Gao G, Jiang L, Guo L, Lin M, Jiao X, Jia W, Huang J (2013). Decreased expression of miR-218 is associated with poor prognosis in patients with colorectal cancer. Int J Clin Exp Pathol.

[28] Zhang C, Guo H, Li B, Sui C, Zhang Y, Xia X, Qin Y, Ye L, Xie F, Wang H (2015). Effects of Slit3 silencing on the invasive ability of lung carcinoma A549 cells. Oncol Rep.

[29] Kim M, Kim JH, Baek SJ, Kim SY, Kim YS (2016). Specific expression and methylation of SLIT1, SLIT2, SLIT3, and miR-218 in gastric cancer subtypes. Int J Oncol.

[30] Shi W, Bastianutto C, Li A, Perez-Ordonez B, Ng R, Chow KY, Zhang W, Jurisica I, Lo KW, Bayley A (2006). Multiple dysregulated pathways in nasopharyngeal carcinoma revealed by gene expression profiling. Int J Cancer.

[31] Narayan G, Goparaju C, Arias-Pulido H, Kaufmann AM, Schneider A, Durst M, Mansukhani M, Pothuri B, Murty VV (2006). Promoter hypermethylation-mediated inactivation of multiple Slit-Robo pathway genes in cervical cancer progression. Mol Cancer.

[32] Dickinson RE, Fegan KS, Ren X, Hillier SG, Duncan WC (2011). Glucocorticoid regulation of SLIT/ROBO tumour suppressor genes in the ovarian surface epithelium and ovarian cancer cells. PLoS One.

[33] Marlow R, Strickland P, Lee JS, Wu X, Pebenito M, Binnewies M, Le EK, Moran A, Macias H, Cardiff RD (2008). SLITs suppress tumor growth in vivo by silencing Sdf1/Cxcr4 within breast epithelium. Cancer Res.

[34] Denk AE, Braig S, Schubert T, Bosserhoff AK (2011). Slit3 inhibits activator protein 1-mediated migration of malignant melanoma cells. Int J Mol Med.

[35] Ng L, Wan TM, Lam CS, Chow AK, Wong SK, Man JH, Li HS, Cheng NS, Pak RC, Cheung AH (2015). Post-operative plasma osteopontin predicts distant metastasis in human colorectal cancer. PLoS One.

[36] Monga SP (2015). Beta-catenin signaling and roles in liver homeostasis, injury, and tumorigenesis. Gastroenterology.

[37] Fiorentino M, Altimari A, D'Errico A, Cukor B, Barozzi C, Loda M, Grigioni WF (2000). Acquired expression of p27 is a favorable prognostic indicator in patients with hepatocellular carcinoma. Clin. Cancer Res.

[38] Chow AK, Ng L, Lam CS, Wong SK, Wan TM, Cheng NS, Yau TC, Poon RT, Pang RW (2013). The enhanced metastatic potential of hepatocellular carcinoma (HCC) cells with sorafenib resistance. PLoS One.

[39] Ng L, Tung-Ping Poon R, Yau S, Chow A, Lam C, Li HS, Chung-Cheung Yau T, Law WL, Pang R (2013). Suppression of actopaxin impairs hepatocellular carcinoma metastasis through modulation of cell migration and invasion. Hepatology.

[40] Noda T, Nagano H, Takemasa I, Yoshioka S, Murakami M, Wada H, Kobayashi S, Marubashi S, Takeda Y, Dono K (2009). Activation of Wnt/beta-catenin signalling pathway induces chemoresistance to interferon-alpha/5-fluorouracil combination therapy for hepatocellular carcinoma. Br J Cancer.

[41] Yang W, Yan HX, Chen L, Liu Q, He YQ, Yu LX, Zhang SH, Huang DD, Tang L, Kong XN (2008). Wnt/beta-catenin signaling contributes to activation of normal and tumorigenic liver progenitor cells. Cancer Res.

[42] Blockus H, Chedotal A (2016). Slit-Robo signaling. Development.

[43] Ikeda S, Kishida S, Yamamoto H, Murai H, Koyama S, Kikuchi A (1998). Axin, a negative regulator of the Wnt signaling pathway, forms a complex with GSK-3beta and beta-catenin and promotes GSK-3beta-dependent phosphorylation of beta-catenin. EMBO J.

[44] Skurk C, Maatz H, Rocnik E, Bialik A, Force T, Walsh K (2005). Glycogen-synthase Kinase3beta/beta-catenin axis promotes angiogenesis through activation of vascular endothelial growth factor signaling in endothelial cells. Circ Res.

[45] Inagawa S, Itabashi M, Adachi S, Kawamoto T, Hori M, Shimazaki J, Yoshimi F, Fukao K (2002). Expression and prognostic roles of beta-catenin in hepatocellular carcinoma: correlation with tumor progression and postoperative survival. Clin. Cancer Res..

[46] Behari J, Zeng G, Otruba W, Thompson MD, Muller P, Micsenyi A, Sekhon SS, Leoni L, Monga SP (2007). R-Etodolac decreases beta-catenin levels along with survival and proliferation of hepatoma cells. J Hepatol.

[47] Delgado E, Bahal R, Yang J, Lee JM, Ly DH (2013). Monga SP: beta-catenin knockdown in liver tumor cells by a cell permeable gamma guanidine-based peptide nucleic acid. Curr Cancer Drug Targets.

[48] Huang SM, Mishina YM, Liu S, Cheung A, Stegmeier F, Michaud GA, Charlat O, Wiellette E, Zhang Y, Wiessner S (2009). Tankyrase inhibition stabilizes axin and antagonizes Wnt signalling. Nature.

[49] Thompson MD, Dar MJ, Monga SP (2011). Pegylated interferon alpha targets Wnt signaling by inducing nuclear export of beta-catenin. J Hepatol.

[50] Schubert T, Denk AE, Ruedel A, Kaufmann S, Hustert E, Bastone P, Bosserhoff AK (2012). Fragments of SLIT3 inhibit cellular migration. Int J Mol Med.

