# Supplementary material for: Suppression of Slit3 induces tumor proliferation and chemoresistance in hepatocellular carcinoma through activation of GSK3β/β-catenin pathway
Source: BMC Cancer. 2018 Jun 1;18:621. doi: 10.1186/s12885-018-4326-5 (PMC5984734; doi:10.1186/s12885-018-4326-5)
Supplement: Supplementary file 1 — Table S1. Slit3 expression and patient characteristics in cohort 1. Table S2. Slit3 expression and patient characteristics in cohort 2. (PDF 78 kb) [file 12885_2018_4326_MOESM1_ESM.pdf]

Additional file 1: Table S1. Slit3 expression and patient characteristics in cohort 1

Patient#	Age	Gender	HBV	Prior liver -directed treatment	Differen-tiation	Cirrhosis	Tumor size	Micro-vascular invasion	Stage	Distant metastasis	Slit3 expression
1	49	M	YES	SURGERY+ RFA/ MICROWAV	MODERATE	NO	7.5	NO	4	Lung	0.516
2	68	M	YES	NO	WELL	NO	7	NO	1	No	3.008
3	65	F	YES	NO	MODERATE	YES	5.5	NO	1	No	1.660
4	38	F	YES	NO	MODERATE	YES	5	YES	2	No	1.515
5	47	F	YES	RESECTION/ RFA	POOR	NO	7	YES	4	Lung, Splenic	1.643
6	67	NA	NA	NO	NA	NA	12	NA	NA	NA	0.518
7	50	F	YES	NO	NA	NO	4	NO	4	Lung, Brain	1.635
8	69	M	YES	NO	MODERATE	YES	17	YES	2	No	0.325
9	34	M	YES	NO	NA	NO	22	NO	1	No	0.833
10	50	M	NA	TOCE	MODERATE	NO	8	NO	1	No	1.142
11	52	M	NO	NO	MODERATE	NO	2.5	NO	3	No	7.797
12	42	M	YES	NO	MODERATE	NO	5	NO	1	No	0.547
13	65	M	NO	NO	WELL	NO	5	NO	1	No	1.627
14	46	M	YES	NO	MODERATE	NO	5.5	NO	1	No	1.579
15	43	M	YES	NO	POOR	YES	8.5	YES	4	Bone, Brain, Lung	1.334
16	64	M	NO	NO	WELL	NO	10	YES	4	Colon, Lung	1.230
17	58	M	YES	NO	NA	NO	7.5	YES	4	Lung	0.655
18	46	M	YES	SURGERY+ RFA/ MICROWAV	WELL	NO	6	NO	1	No	6.126
19	55	M	NA	NO	WELL	NO	15	YES	4	Brain, Lung	0.568
20	41	NA	NA	NO	NA	YES	9	NA	NA	NA	0.232
21	53	M	YES	NO	WELL	YES	4.8	NO	1	No	1.798
22	71	F	NA	NO	NA	NO	10	YES	2	No	0.804
23	56	M	YES	NO	WELL	NO	6.5	NO	1	No	6.139
24	47	M	YES	NO	POOR	YES	14	NO	4	Lung, Liver	1.243
25	54	M	YES	NO	NA	NO	5	YES	4	Lung	9.455
26	56	M	YES	NO	MODERATE	NO	17	YES	4	Lung, Adrenal	1.638
27	43	M	YES	NO	MODERATE	NO	8	YES	4	Lung, Brain	0.579
28	48	M	YES	TOCE	MODERATE	NO	17	NO	3	No	0.026
29	67	M	YES	NO	NA	NO	6	NO	4	Lung	0.272
30	68	M	NA	NO	MODERATE	NO	10	YES	3	Lymph node	0.474
31	64	M	YES	NO	NA	NO	4	NO	1	No	1.165
32	29	M	YES	NO	MODERATE	NO	3.8	NO	1	No	1.248
33	51	M	YES	NO	MODERATE	YES	8	NO	1	No	0.603
34	44	M	YES	NO	WELL	NO	7	NO	1	No	0.756
35	49	M	YES	NO	NA	YES	4	NO	4	Pleura, Lung	1.828
36	64	M	YES	NO	MODERATE	NO	13	YES	4	Lung, Bone, Peritoneal	0.516
37	42	M	NO	NO	MODERATE	NO	11	YES	2	No	1.975
38	60	M	YES	NO	NA	YES	20	NO	1	No	1.175
39	52	M	NO	NO	MODERATE	YES	2.9	YES	4	Lung	0.062
40	63	F	YES	NO	MODERATE	YES	4.5	NO	1	No	1.927

Additional file 1: Table S2: Slit3 expression and patient characteristics in cohort 2

Patient#	Age	Gender	Tumor size	Slit3 expression
1	65	M	13	2.336
2	82	M	10	0.596
3	79	M	7.5	4.441
4	65	M	4.5	0.252
5	77	M	3.5	6.342
6	60	M	14	0.189
7	65	F	1.9	1.098
8	71	M	6	0.5724
9	60	M	11	0.3035
10	60	M	2.8	1.5337
11	66	M	8	1.9656
12	65	F	8.5	1.95E-03
13	57	M	2	1.2346
14	55	M	15	0.2242
15	67	M	10.5	0.4993
16	68	M	9.5	0.546
17	57	F	8	1.1219
18	63	F	2.5	1.8063
19	61	M	2.5	0.9924
20	55	M	4	0.463
21	46	F	17	0.4503
22	47	M	14	0.568
23	78	M	10	0.5897
24	55	M	7.5	1.3168
25	41	F	6.5	0.1635
